# THz‐Wave Absorption Properties of Organic–Inorganic Hybrid Perovskite Materials: A New Candidate for THz Sensors

**DOI:** 10.1002/smsc.202300186

**Published:** 2024-01-15

**Authors:** Inhee Maeng, Young Mi Lee, Min-Cherl Jung

**Affiliations:** ^1^ YUHS-KRIBB Medical Convergence Research Institute College of Medicine Yonsei University Seoul 03722 Republic of Korea; ^2^ Beamline Department Pohang Accelerator Laboratory POSTECH Pohang 37673 Republic of Korea; ^3^ Division of Materials Science Institute of Pure and Applied Sciences University of Tsukuba Ibaraki 305-8577 Japan

**Keywords:** organic–inorganic hybrid perovskite materials, phonon vibrational modes, THz absorptions, THz sensors

## Abstract

Over the past two decades, organic–inorganic hybrid perovskite materials (OHP) have been extensively explored across various scientific disciplines, including physics, chemistry, and materials science, with a primary focus on solar cells. Building on numerous studies, the development of OHP‐based solar cells has transitioned into practical product realization, instilling the anticipation of novel solar cell advancements. Notably, OHP demonstrates versatility beyond its conventional application in solar cell materials. The physical properties of OHP materials exhibit a unique signature, thereby underscoring their potential utility as innovative functional materials, encompassing light‐emitting diodes, lasers, and photodetectors. Recent reports on terahertz (THz)‐wave absorption properties of OHP materials indicate a high possibility of their potential application as THz sensors. From the viewpoint of medical devices, which hold the most promising application potential, the exploration of optical phonon vibrational modes in the 0.5–3 THz frequency range is important. Moreover, understanding the correlations between atomic structure and lattice vibration modes is indispensable. In this concise review, the THz‐wave absorption properties exhibited by 3D OHP materials are meticulously explored. Furthermore, future research directions for THz sensors using OHP materials are suggested.

## Graphical abstract

Recent reports on terahertz (THz)‐wave absorption properties of OHP materials indicate a high possibility of their potential application as THz sensors. From the viewpoint of medical devices, the exploration of optical phonon vibrational modes in the 0.5–3 THz frequency range is essential. In this concise review, the THz‐wave absorption properties exhibited by 3D OHP materials are meticulously explored.

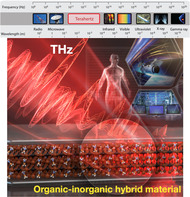

## 1 Introduction

Over the past two decades, research on solar cell applications using organic–inorganic hybrid perovskite (OHP) materials has yielded remarkable outcomes, positioning the field closer to commercial production.^[^ ^1^, ^2^, ^3^, ^4^, ^5^, ^6^, ^7^, ^8^, ^9^, ^10^, ^11^, ^12^, ^13^, ^14^, ^15^, ^16^, ^17^ ^]^ OHP‐based solar cells exhibit an efficiency of 25.8% as single cells, surpassing conventional solar cells based on silicon (25.4%), CdTe (22.1%), and copper indium gallium selenide (CIGS).^[^ ^2^, ^12^, ^18^ ^]^ Despite the favorable viewpoint of product realization, the solar cell application is still ongoing research because of material instability, halogen ion migration, and short device lifetime.^[^ ^18^, ^19^, ^20^, ^21^, ^22^, ^23^, ^24^, ^25^, ^26^, ^27^, ^28^, ^29^ ^]^ These adverse properties are being addressed through subsequent research, and ultimately, there is widespread anticipation of the imminent achievement of commercialization.^[^ ^29^, ^30^ ^]^

OHP materials became significant in 2009 when Miyasaka and co‐workers implemented them as solid active materials in dye‐sensitized solar cell architectures, sparking interest in their applications.^[^ ^31^ ^]^ Fundamental studies which emerged from this result revealed their potential utility as solar cell materials because of their: 1) broad absorption photon range (≈800 nm), 2) exceedingly weak exciton binding energy (≈0.03 eV), and 3) rapid charge carrier transport (7.5 and 12.5 cm^2^ V^−1^ s^−1^ for electrons and holes, respectively).^[^ ^32^, ^33^, ^34^, ^35^ ^]^

In addition to solar cells, the remarkable properties of these hybrid perovskite materials have led to diverse possibilities in their applications, including light‐emitting diodes (LED), lasers, and photodetectors.^[^ ^36^, ^37^, ^38^, ^39^, ^40^, ^41^, ^42^, ^43^, ^44^, ^45^, ^46^, ^47^, ^48^, ^49^, ^50^ ^]^ Particularly in LEDs, easy bandgap engineering through halogen element substitution has demonstrated the potential for full‐color displays.^[^ ^51^, ^52^ ^]^ Rapid material degradation, however, has not yet been addressed. The pursuit of material stability remains a crucial focus, particularly regarding device lifetime for LED applications.

**Figure 1:**
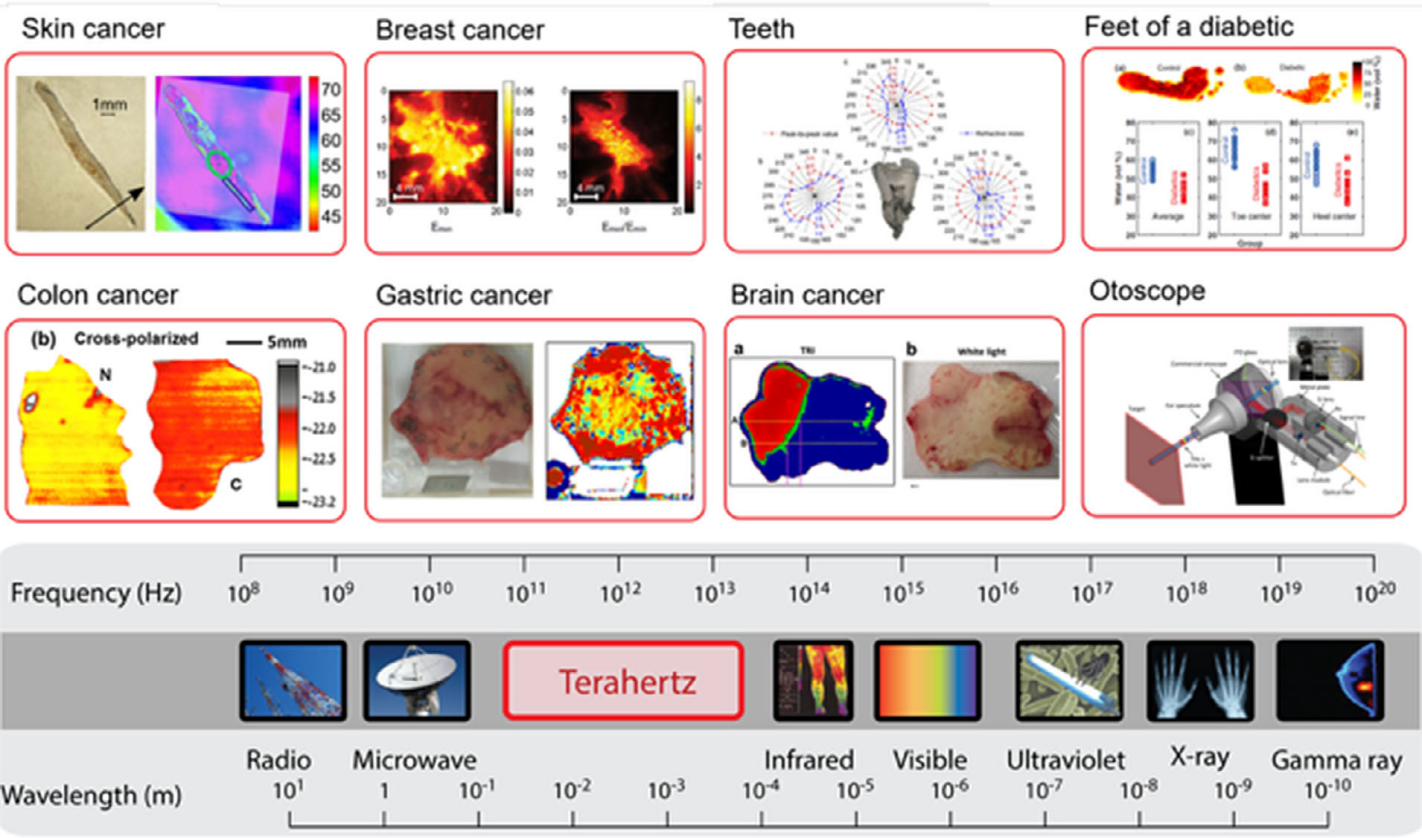
Terahertz sensing applications based on the physical sensitivity of THz‐wave. Images for “Skin cancer”: Reproduced under the terms of the CC‐BY Creative Commons Attribution 4.0 International license (https://creativecommons.org/licenses/by/4.0).^[^ ^87^ ^]^ Copyright 2023, published by Optica Publishing Group. Images for “Breast cancer”: Reproduced with permission.^[^ ^88^ ^]^ Copyright 2009, Optica Publishing Group. Images for “Teeth”: Reproduced with permission.^[^ ^89^ ^]^ Copyright 2022, Optica Publishing Group. Images for “Feet of a diabetic”: Reproduced under the terms of the CC‐BY Creative Commons Attribution 4.0 International license (https://creativecommons.org/licenses/by/4.0).^[^ ^90^ ^]^ Copyright 2022, The Authors, published by Springer Nature. Images for “Colon cancer”: Reproduced under the terms of the CC‐BY Creative Commons Attribution 4.0 International license (https://creativecommons.org/licenses/by/4.0).^[^ ^91^ ^]^ Copyright 2015, The Authors, published by SPIE. Images for “Gastric cancer”: Reproduced with permission.^[^ ^92^ ^]^ Copyright 2015, Optical Society of America. Images for “Brain cancer”: Reproduced under the terms of the CC‐BY Creative Commons Attribution 4.0 International license (https://creativecommons.org/licenses/by/4.0).^[^ ^93^ ^]^ Copyright 2014, The Authors, published by Springer Nature. Images for “Otoscope”: Reproduced with permission.^[^ ^94^ ^]^ Copyright 2016, Optical Society of America.

Due to their favorable physical properties, OHPs are being explored as active materials in various applications, with particular interest in THz sensors.^[^ ^46^, ^53^, ^54^, ^55^, ^56^, ^57^, ^58^, ^59^, ^60^ ^]^ Theoretical analyses of the atomic structure of OHP materials predict phonon vibrational modes encompassing frequencies that are suitable for lattice vibration (1–100 THz), molecular vibration (2 THz), and molecular rotation (0.3 THz).^[^ ^61^ ^]^ The 1–100 THz range associated with lattice vibrations is suitable for typical THz sensor applications. OHPs with low unit cost, simple fabrication process, flexibility, and potential for large‐area film fabrication are promising in medical device applications that require high sensitivity for detecting molecules within the human body (**Figure** **1**).^[^ ^53^, ^62^, ^63^, ^64^, ^65^, ^66^, ^67^, ^68^, ^69^, ^70^, ^71^ ^]^ Due to the fundamental physical properties of OHP materials, with a typical AMX_3_ crystal structure, the ease of substitution among organic (or inorganic) cations (A: CH_3_NH_3_^+^ (MA), HC(NH_2_)_2_^+^ (FA), or Cs^+^), metal cations (M: Pb^+^ or Sn^+^), and halogen anions (X: Cl^−^, Br^−^, or I^−^) enables the creation of a variety of phonon vibrational modes within the 0.3–100 THz range. Furthermore, the investigation of phonon vibration modes of OHP materials within the 0.5–3.0 THz range which is sensitive to molecules in the human body should be conducted (Figure 1).

This review aims to consolidate the reported research from this perspective, discuss the intriguing THz absorption properties at room temperature (RT), and suggest pathways for realizing THz sensors using OHP materials.

## 2 THz‐Wave Properties in MAPbI_3_ and MAPbBr_3_ Thin Films

### 2.1 Defect‐Incorporated Structure and Its Significant THz Absorption Property (MAPbI_3_)

**Figure 2:**
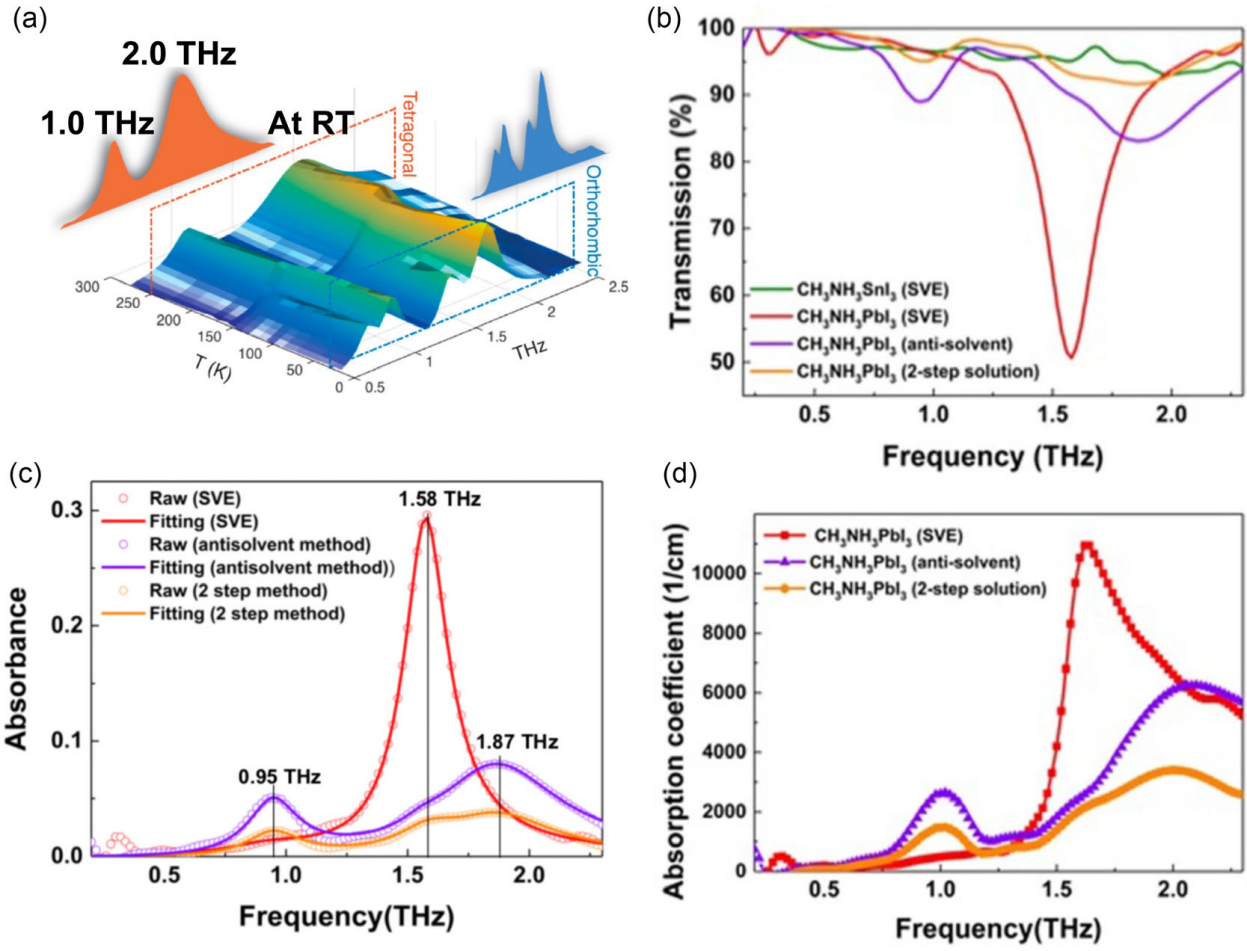
a) THz‐wave absorption properties of MAPbI_3_ thin film (solution prepared) at various temperatures. Reproduced with permission.^[^ ^72^ ^]^ Copyright 2016, American Chemical Society. b) THz‐wave absorption properties of MAPbI_3_ (vacuum evaporated) at RT. c) Different absorption peaks for various fabrication methods. d) In the MAPbI3 fabricated using the SVE method, a remarkable absorption coefficient exceeding 10 000 cm^−1^ is observed. b–d) Reproduced under the terms of the CC‐BY Creative Commons Attribution 4.0 International license (https://creativecommons.org/licenses/by/4.0).^[^ ^74^ ^]^ Copyright 2019, The Authors, published by Springer Nature.

In 2016, La‐O‐Vorakiat et al. performed THz‐time‐domain spectroscopy (THz‐TDS) experiments using a representative OHP thin film composed of MAPbI_3_.^[^ ^72^ ^]^ They observed two phonon modes at 1 and 2 THz originated from the buckling of Pb–I–Pb angles and the Pb–I length vibrations, respectively (**Figure** **2**a). In the 0.5–2.5 THz, notably, there is no report for the phonon mode originating from the CH_3_NH_3_ cation. Consistently, A. M. A. Leguy, et al. found no molecular‐based phonon mode confirmed by Raman spectroscopy experiment and theoretical calculation.^[^ ^73^ ^]^ Their results suggested the absence of phonon modes originating from the organic cations in the 0.5–3.0 THz range, a key area for the THz sensor.

In 2019, Maeng, et al. reported additional THz absorption features in MAPbI_3_ (Figure 2b).^[^ ^74^ ^]^ A THz absorption peak was observed at 1.58 THz with a significant absorption coefficient of ≈50%. Interestingly, the different observations between two studies were attributed to the difference in fabrication methods. While OHP thin films are often produced using solution‐prepared methods (which differs from the antisolvent method), the study conducted by Maeng, et al employed a sequential vacuum evaporation (SVE) method.^[^ ^25^, ^75^, ^76^, ^77^ ^]^ These two methods exhibit significant differences in the properties of thin films. First, thin‐film fabrication using the SVE method shows smaller grain sizes compared to the solution‐prepared methods, therefore resulting in higher grain boundary densities. Second, the increase in grain size results in an increased density of molecular defects, such as CH_3_NH_2_ molecules (Figure 2c). The high density of CH_3_NH_2_ molecular defects contributes to the formation of CH_3_NH_2_‐incorporated perovskite structure and leads to the giant 1.58 THz absorption with an absorption coefficient exceeding 10 000 cm^−1^ (Figure 2d).

**Figure 3:**
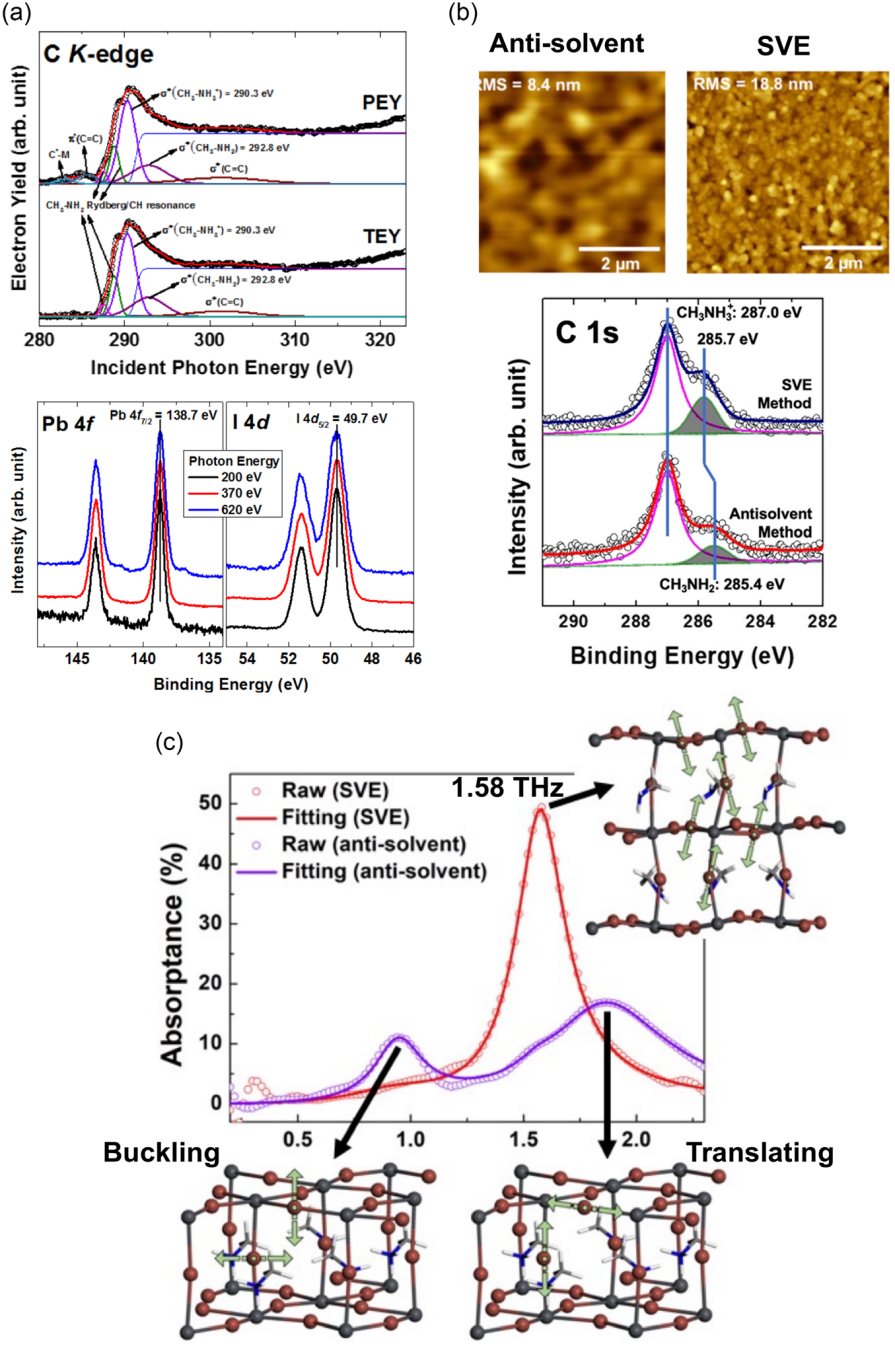
a) C *K*‐edge absorption and core‐level spectra of Pb 4f and I 4d measured by near‐edge X‐Ray absorption of fine structure and high‐resolution XPS using synchrotron radiation. The chemical state of the CH_3_NH_2_ defect is observed in the C *K*‐edge in both partial electron yield and total electron yield modes. Notably, the chemical states of the Pb 4f and I 4d core‐levels are unchanged. Reproduced under the terms of the CC‐BY Creative Commons Attribution 4.0 International license (https://creativecommons.org/licenses/by/4.0).^[^ ^24^ ^]^ Copyright 2016, The Authors, published by AIP Publishing. b) The differences in the surface morphology and C 1s chemical state are dependent on to the fabrication method. c) MAPbI_3_ fabricated using the SVE method shows a new vibrational mode at 1.58 THz which originated from CH_3_NH_2_ defect‐incorporated perovskite structure. b,c) Reproduced under the terms of the CC‐BY Creative Commons Attribution 4.0 International license (https://creativecommons.org/licenses/by/4.0).^[^ ^74^ ^]^ Copyright 2019, The Authors, published by Springer Nature.

Interestingly, the CH_3_NH_2_ molecular defect was first discovered through synchrotron radiation‐based experiments in MAPbI_3_ thin films fabricated using a solution‐prepared method (**Figure** **3**a).^[^ ^24^ ^]^ The chemical states of Pb and I with CH_3_NH_2_ molecular defects did not differ from the original chemical state of the OHP, which demonstrates the equivalence of the chemical states of Pb and I with CH_3_NH_3_^+^. Based on these results, CH_3_NH_2_ molecular defects were determined to be located at the grain boundaries. Importantly, these molecular defects induced a unique phonon mode at 1.58 THz, exhibiting significant THz absorption properties (Figure 3b,c).

**Figure 4:**
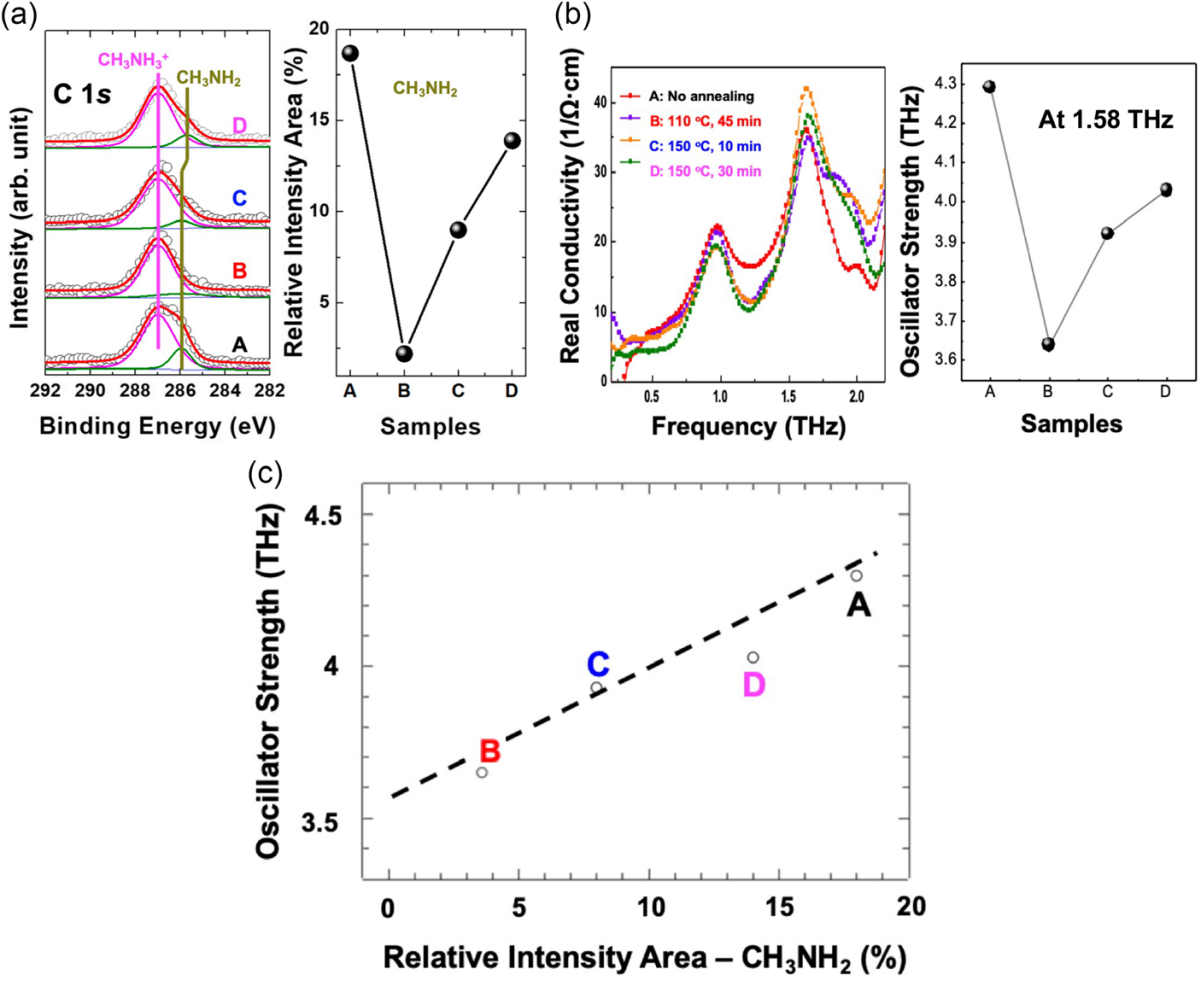
a) Carbon 1s core‐level spectra and the relative intensity area (%) of CH_3_NH_2_ defect. b) THz‐wave absorptions and the oscillator strength (Ω^−1^ cm^−1^) at 1.58 THz. c) Linear correlation can be established between the concentration of CH_3_NH_2_ defects and oscillator strength at 1.58 THz. a–c) Reproduced under the terms of the CC‐BY Creative Commons Attribution 4.0 International license (https://creativecommons.org/licenses/by/4.0).^[^ ^57^ ^]^ Copyright 2020, The Authors, published by MDPI.

To further demonstrate this significant absorption property, Maeng et al. conducted a study in 2020 to investigate the correlation between the density of CH_3_NH_2_ defects and the 1.58 THz absorption peak.^[^ ^57^ ^]^ (Figure 3c) Under various postannealing conditions, the density of CH_3_NH_2_ molecular defects obtained from the C 1s core‐level spectra showed correlation with the THz absorption (**Figure** **4**a). A linear correlation between the density of the CH_3_NH_2_ molecular defects and the THz oscillator strength at 1.58 THz was confirmed (Figure 4b), revealing that higher densities of the CH_3_NH_2_‐incorporated perovskite structures led to significantly increased oscillator strengths (Figure 4c). Paradoxically, in the context of THz sensor applications, controlling defect densities is pivotal for inducing THz absorption. However, the high density of defects negatively affects material stability and presents challenges to the lifetime of a device. Consequently, THz sensor applications for MAPbI_3_ are limited.

### 2.2 Nonaffected Defect Structure and Its THz Absorption Property (MAPbBr_3_)

The lattice constant of OHP materials can be tuned through the substitution of halogen elements. The focus of this review is the 0.5–3.0 THz phonon vibrational modes in OHP materials, which are attributed to the lattice constant between the metal cation and halogen anion. Elements such as I, Br, and Cl, contained within the same group in the periodic table, can be easily substituted through fabrication processes to yield OHP materials like MAPbI_3_ (Cubic: 6.31 Å), MAPbBr_3_ (5.99 Å), and MAPbCl_3_ (5.67 Å). Notably, the tolerance factor (*t*) of MAPbBr_3_ (*t* = 0.83) is higher than that of MAPbI_3_ (*t* = 0.81), making MAPbBr_3_ more stable.^[^ ^78^, ^79^, ^80^ ^]^

**Figure 5:**
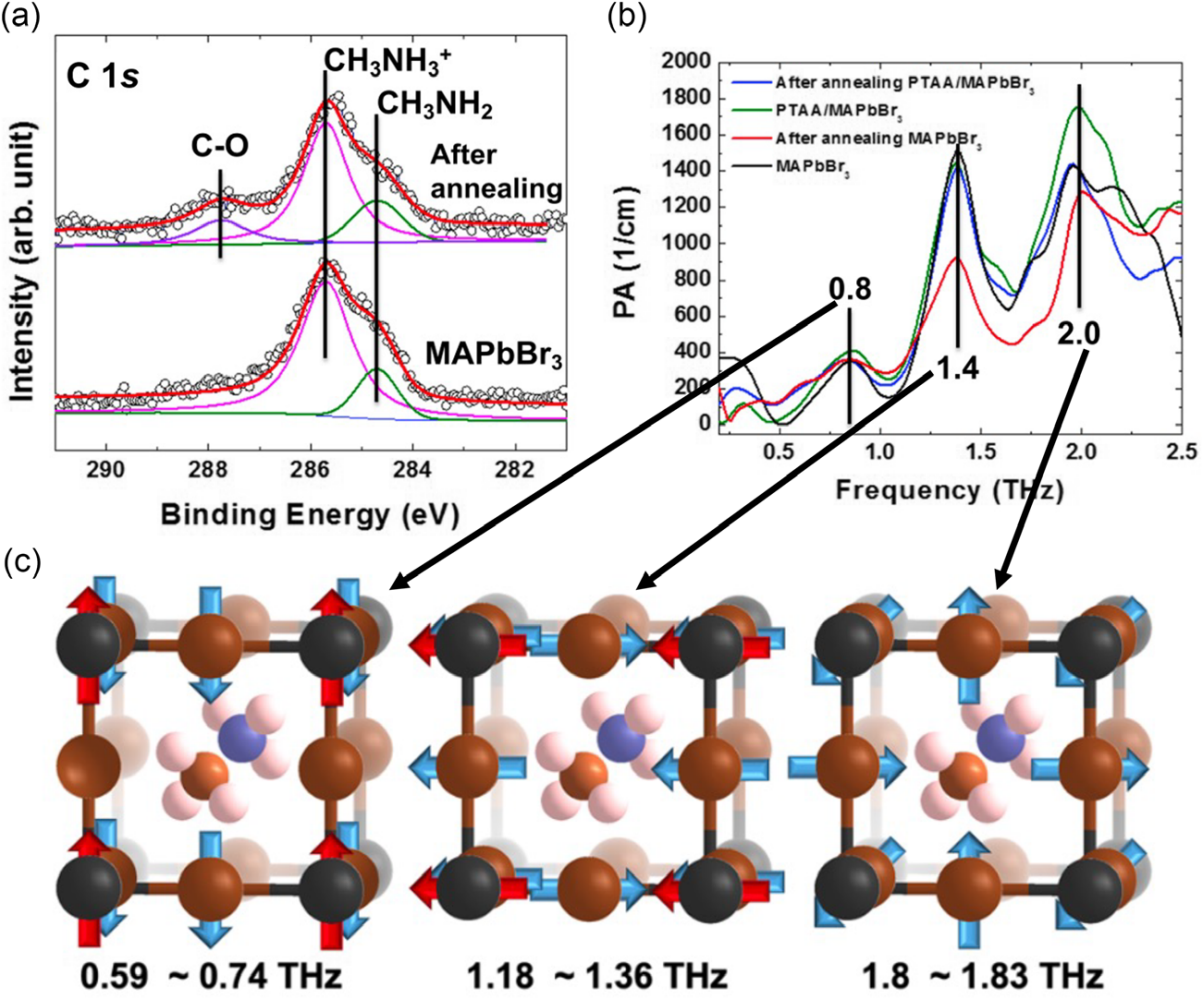
a) Carbon 1s core‐level spectra before and after postannealing. The CH_3_NH_2_ defect and C–O surface contamination are observed. b) THz‐wave absorption of MAPbBr_3_ thin film. c) Three absorption peaks at 0.8 (buckling), 1.4 (translating), and 2.0 (Br self‐vibration) THz are observed. a–c) Reproduced under the terms of the CC‐BY Creative Commons Attribution 4.0 International license (https://creativecommons.org/licenses/by/4.0).^[^ ^55^ ^]^ Copyright 2020, The Authors, published by Springer Nature.

Thin films of MAPbBr_3_ fabricated via the SVE method exhibit distinctly different THz absorption properties compared to MAPbI_3_ thin films (**Figure** **5**).^[^ ^55^ ^]^ The observed chemical states at the C 1s core level appear not only as CH_3_NH_2_ molecular defects but also as C–O contaminants (Figure 5a). These different chemical states could provide a variety of phonon modes in THz absorption property. Unfortunately, these expected phonon modes are not observed to be the origin of any THz absorption (Figure 5b). Despite various postannealing treatments, three phonon vibration modes at 0.8, 1.4, and 2.0 THz were consistently observed; however, the maximum power absorption (PA) did not exceed 2000 cm^−1^ (Figure 5b). The modes at 0.8, 1.4, and 2.0 THz confirmed transverse, longitudinal optical, and Br self‐vibrational modes, respectively (Figure 5c). Remarkably, the Br self‐vibrational mode was observed in MAPbBr_3_, but not in the I halogen of MAPbI_3_.

From these results, we can determine the distinct effects of Br compared to I on the THz absorption property. First, studies with Br indicated that defects within the thin film have almost no impact on the THz absorption properties. Specifically, the CH_3_NH_2_ molecular defect located at the grain boundaries and C–O contaminants on the surface of MAPbBr_3_ thin film do not influence its THz absorption properties. Second, the introduction of Br results in a new self‐vibrational mode, thus demonstrating that the vibrational effect of the Br halogen itself is greater than that of the defect‐incorporated perovskite structure with the I halogen elements. Third, the insertion of the Br halogen element leads to a lattice constant reduction of ≈0.32 Å, resulting in absorption peaks at a lower frequency relative to the THz absorption in MAPbI_3_. For instance, because of the same underlying causes (buckling and translating in Pb–X–Pb: X = halogen such as I or Br), the 1.0 and 2.0 THz absorption peaks observed in MAPbI_3_ manifest as 0.8 and 1.4 THz absorption peaks in MAPbBr_3_, respectively. From the perspective of the exchange of organic cations, one can intuitively expect that formamidinium (FA)‐based hybrid perovskites, such as α‐FAPbI_3_ (black perovskite, cubic structure) with a lattice constant of ≈6.36 Å, may yield THz absorption at even higher frequencies compared to those observed in MAPbI_3_.^[^ ^81^ ^]^ This comparison referenced by the lattice constant will be discussed later. The THz absorption property observed in MAPbBr_3_ thin films shows fixed THz absorptions, irrespective of the presence of defects. However, the PA threshold of 2000 cm^−1^ is a limiting factor.

In addition to the unique THz absorption property in MAPbBr_3_, this study presents the possibility for flexible and stable THz sensor fabrication through the incorporation an ultrathin protection layer using a polymer [poly[bis(4‐phenyl)(2,4,6‐trimethylphenyl)amine]] (PTAA) and a flexible substrate (PET) to achieve THz transmittance exceeding 90%.^[^ ^58^ ^]^ The introduction of a protective layer and flexible substrate may be suitable applications as medical devices.

## 3 THz‐Wave Absorption Properties in FAPbI_3_ Thin Film and Single Crystal

**Figure 6:**
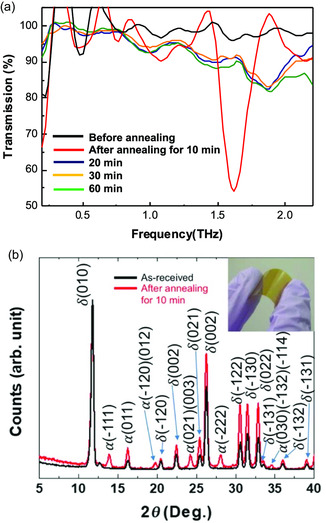
a) The unusual THz‐wave absorption at 1.62 THz is observed in the sample after postannealing for 10 min. b) This sample has the δ/α‐mixed phase, which was confirmed by X‐ray diffraction (XRD) measurements. a,b) Reproduced with permission.^[^ ^75^ ^]^ Copyright 2019, The Japan Society of Applied Physics.

In 2019, Lee et al. reported on the THz absorption property of FAPbI_3_ thin films fabricated using the SVE method.^[^ ^75^ ^]^ They observed significant THz absorption peaks when the mixed phase with two atomic phases (*δ*‐ and *α*‐phases) was formed (**Figure** **6**a). Before and after annealing, the *δ*‐ and *α*‐phase comprised the atomic phase of FAPbI_3_, respectively (Figure 6b). Interestingly, after being subjected to a 10 min. postannealing period, the sample exhibited a significant THz absorption peak at 1.62 THz which was characterized by transmittance exceeding 40%. Unfortunately, this report does not mention the detailed discussion of the origin of the *δ*/*α*‐mixed phase. This is because of the limitations of thin films (<300 nm), including grains, which can provide many factors such as defects, and the use of simplistic chemical state analysis to understand the origin of phonon vibration modes. The origin of this unusual THz absorption property in the *δ*/*α*‐mixed FAPbI_3_ can be assumed in two ways: first, it could be due to defect‐incorporated perovskite structure like those observed in MAPbI_3_; second, it could arise from a unique structure at the interface between *δ*‐ and *α*‐phases. To elucidate the origin of this unusual THz absorption property, physical properties associated with thin films, such as grain and grain boundary, must be eliminated by transitioning from thin films to single crystals.

**Figure 7:**
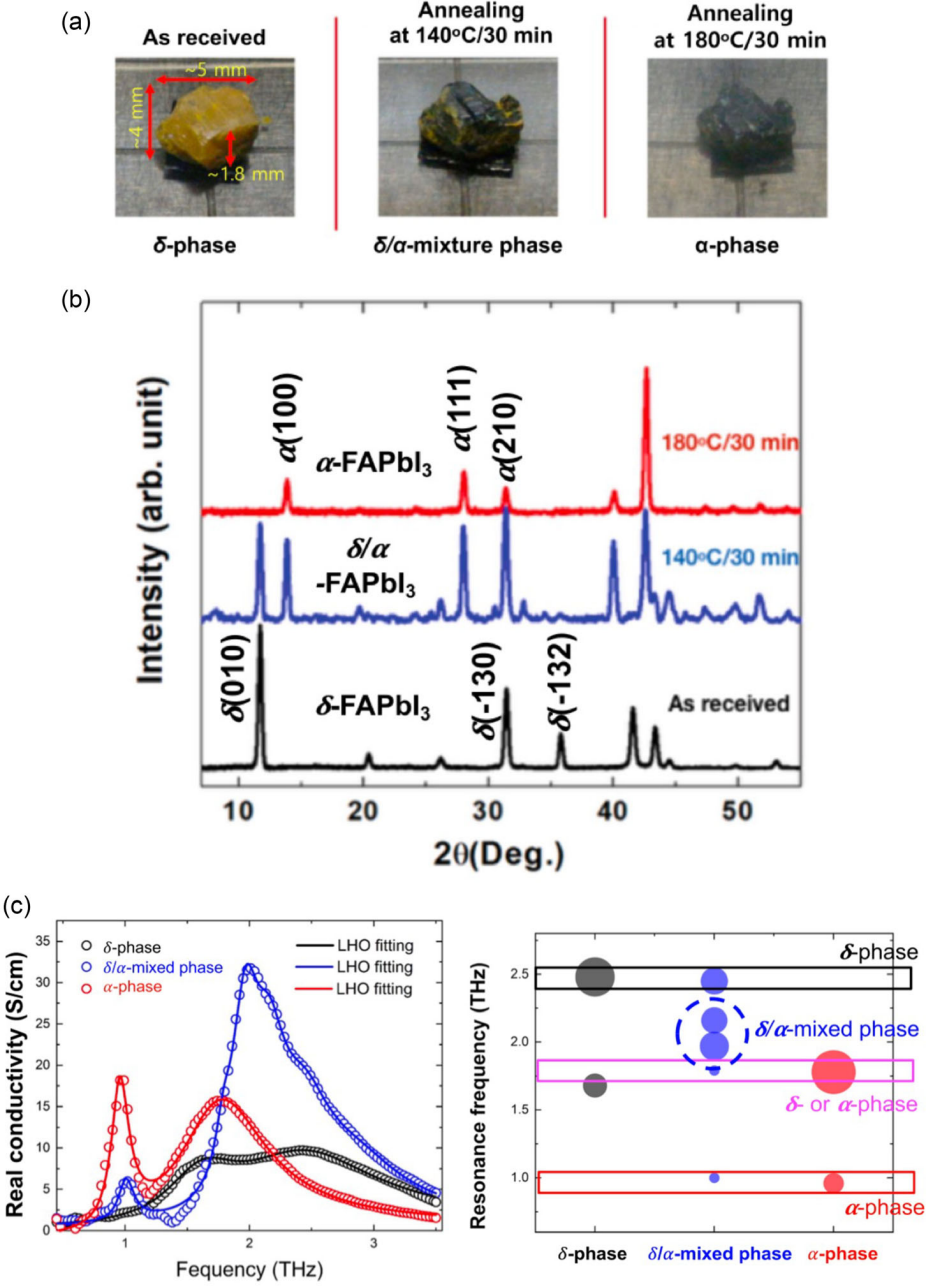
a,b) The *δ‐, δ/α*‐mixture, and α‐phase (confirmed by XRD measurements) of three different colors formed through postannealing. c) THz‐wave absorptions of each phase. The unique THz‐wave absorption is observed in the *δ*/*α*‐mixed phase. a–c) Reproduced under the terms of the CC‐BY Creative Commons Attribution 4.0 International license (https://creativecommons.org/licenses/by/4.0).^[^ ^58^ ^]^ Copyright 2021, The Authors, published by Springer Nature.

**Figure 8:**
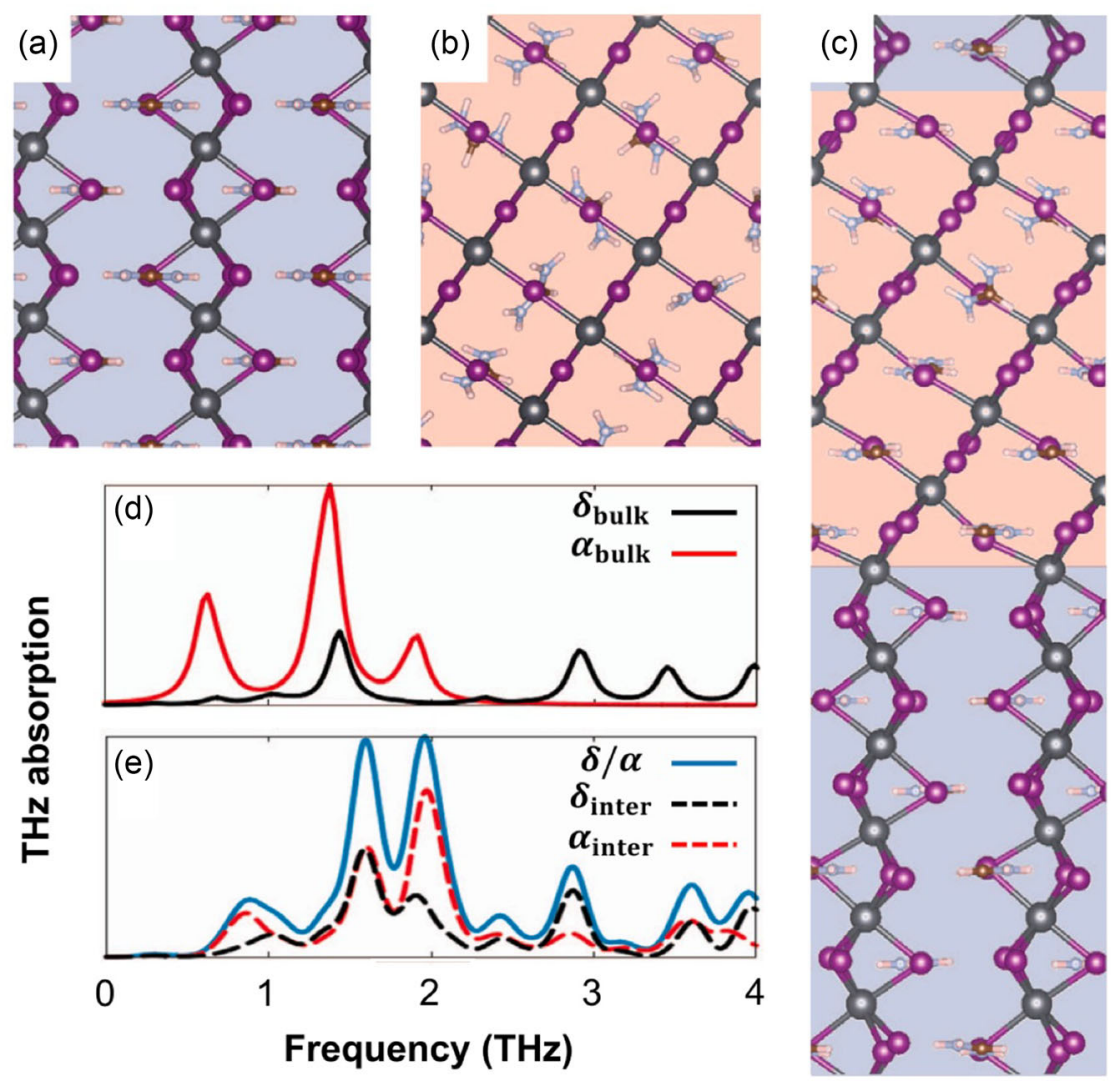
a–c) The atomic structures of *α*‐phase (a), *δ*‐phase (b), and *δ*/*α*‐mixed phase (c). In the structural energy calculation, the *δ*/*α*‐mixed phase has no interface. d,e) In the theoretical simulation, the interfacial phonon vibrational mode appears at frequencies which match the experimental data. a–e) Reproduced under the terms of the CC‐BY Creative Commons Attribution 4.0 International license (https://creativecommons.org/licenses/by/4.0).^[^ ^58^ ^]^ Copyright 2021, The Authors, published by Springer Nature.

In 2021, Maeng, et al. prepared single crystal FAPbI_3_ and generated *δ*‐, *δ*/*α*‐mixed, and *α*‐phases through simple postannealing (**Figure** **7**a,b).^[^ ^58^ ^]^ Similar to previous research, they observed the unusual THz absorption property in the *δ*/*α*‐mixed phase through a THz‐TDS experiment (Figure 7c). The consistent observation of the unusual THz absorption peak provides clear evidence that the phonon vibrational mode does not originate from the defect‐incorporated perovskite structure, thus indicating a different origin from that observed for MAPbI_3_. Because they selected the single crystal which confirmed no defect‐incorporated structure. Interestingly, unusual THz absorption peaks at 2.0 and 2.2 THz unique to the *δ*/*α*‐mixed phase were observed; these contained peaks present in both the *δ*‐ and *α*‐phase (Figure 7c). The theoretical simulation confirms that these two unusual THz absorptions originated from the interface of two different atomic phases (**Figure** **8**). Consequently, the unusual THz absorption property observed in the *δ*/*α*‐mixed phase of FAPbI_3_ arises from interfacial phonon vibrations unique to the interface of the *δ*‐ and *α*‐phase.

Unfortunately, this study did not quantitatively explore the density of interfaces between the *δ*‐ and *α*‐phase that would optimize the interfacial phonon vibrational modes. If subsequent research is performed with a different ratios of 1:3, 1:1, and 3:1 of the δ/α‐mixed phase, it would confirm a trend of interfacial phonon vibration modes.

Initially, the motivation of this study was to explore the possibility of modulating THz absorption frequencies by fine tuning the lattice constant (6.31→ 6.36 Å) through the substitution of the organic cation (MA → FA) to investigate the influence of the atomic phase of FAPbI_3_ on unusual THz absorption. However, the presence of unusual THz absorption features originating from the mixed phase in FAPbI_3_ suggests the potential presence of a unique THz absorption property present not only in typical 3D hybrid perovskites, but also in 2D hybrid perovskites (Pb free) such as MA_3_Sb_2_I_9_ and MA_3_Bi_2_I_9_.

## 4 THz‐Wave Properties in Solution‐Prepared γ‐ and δ‐CsPbI_3_

**Figure 9:**
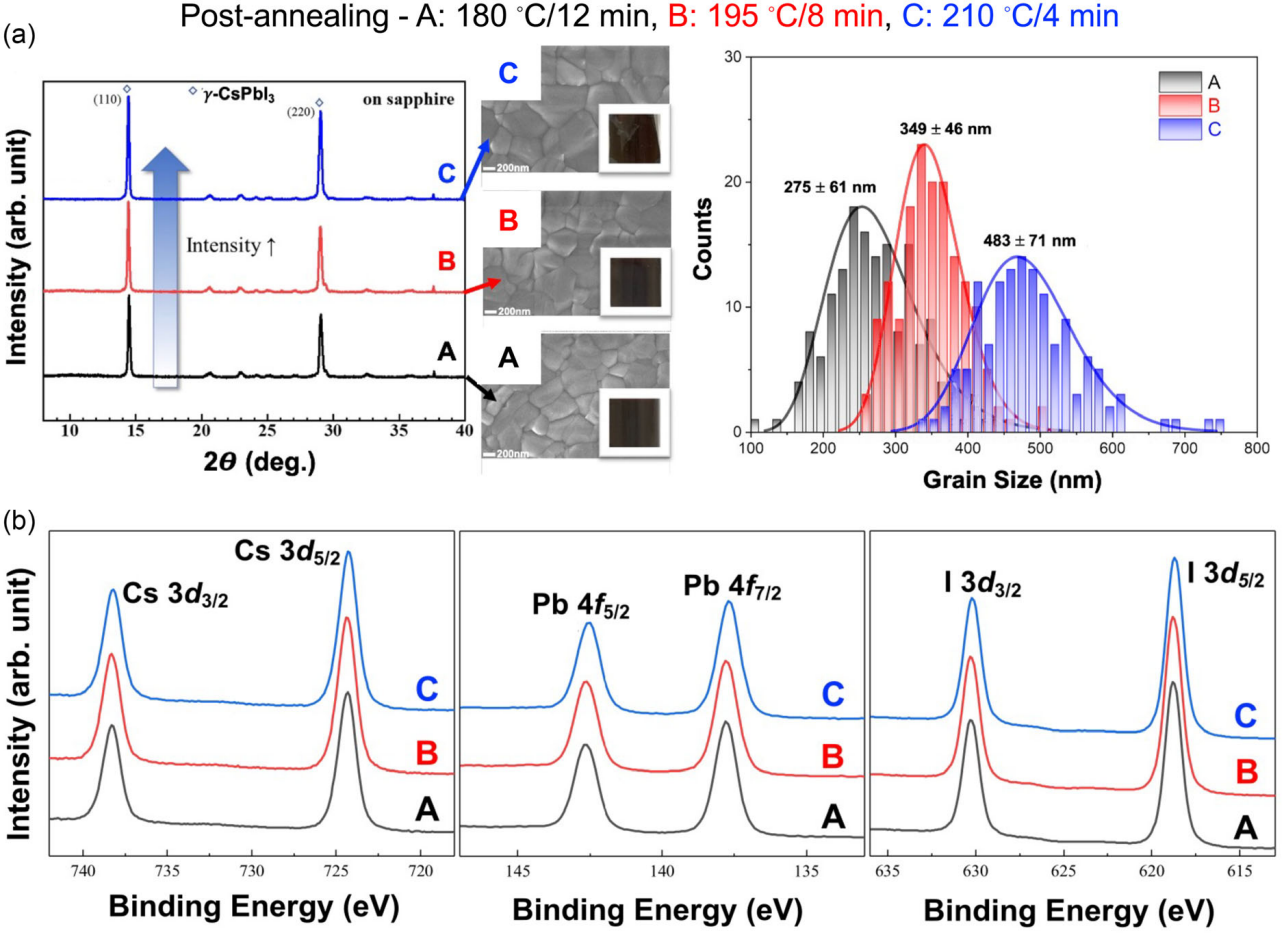
a) The atomic structures and surface morphologies measured by XRD and atomic force microscopy are shown in *γ*‐CsPbI_3_ of different grain sizes. b) Interestingly, there is no change in the chemical states of Cs 3d, Pb 4f, and I 3d core‐level spectra. a,b) Reproduced under the terms of the CC‐BY Creative Commons Attribution 4.0 International license (https://creativecommons.org/licenses/by/4.0).^[^ ^60^ ^]^ Copyright 2023, The Authors, published by Elsevier.

Finally, we discuss the THz absorption property of all‐inorganic perovskite CsPbI_3_, where the organic cation is replaced by an inorganic cation (Cs). A recent report investigated the THz absorption properties of *γ*‐CsPbI_3_, fabricated by the solution‐prepared method, with various grain boundary sizes created using various postannealing conditions (**Figure** **9**a).^[^ ^60^ ^]^ Interestingly, *γ*‐CsPbI_3_ samples fabricated with average grain sizes of 275, 349, and 483 nm, each, exhibit consistent optical bandgaps and chemical states, including the Cs 3d, Pb 4f, and I 3d core levels (Figure 9b). This shows the same atomic and electronic structures of the films without any defects.

**Figure 10:**
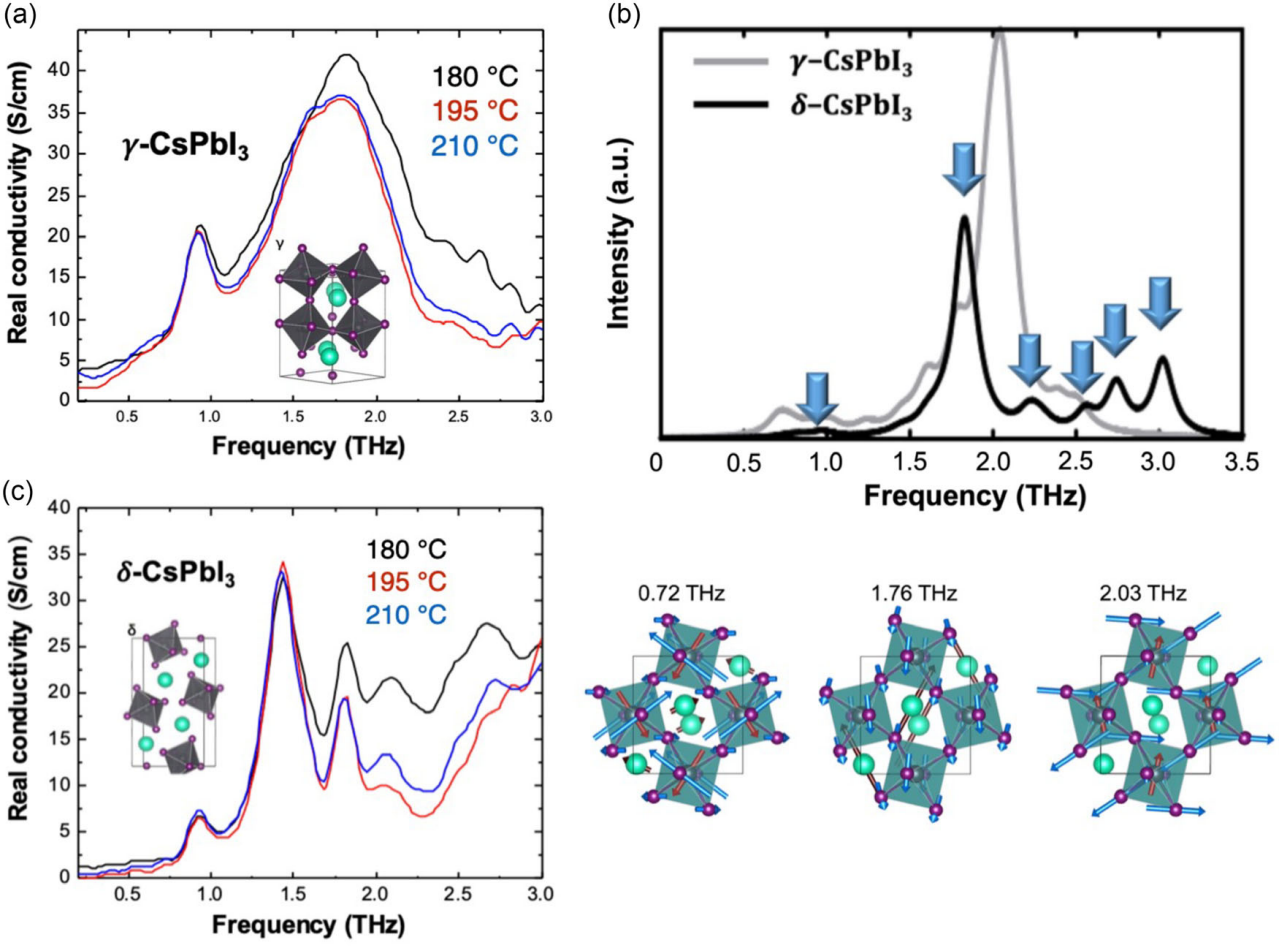
a–c) The *γ*‐CsPbI_3_, *δ‐*CsPbI_3,_ and theoretical simulation results. A wide THz‐wave absorption feature in the range of 0.5–3.0 THz is observed. a–c) Reproduced under the terms of the CC‐BY Creative Commons Attribution 4.0 International license (https://creativecommons.org/licenses/by/4.0).^[^ ^60^ ^]^ Copyright 2023, The Authors, pubslished by Elsevier.

Remarkably, THz absorption peaks are observed across the entire range of 0.5–3 THz, with a relative conductivity that approaches a maximum of 40 S cm^−1^ (**Figure** **10**a). Furthermore, samples with different grain sizes show no significant differences in THz absorption properties. In the case of CsPbI_3_, this is because of the absence of defects and the substitution of organic cations with inorganic Cs cations, which induce THz absorption throughout the 0.5–3 THz range (Figure 10a). From the theoretical simulation, they confirmed that the observed 0.9, 1.5, and 1.8 THz originated from the transverse I–Pb–I frame, Cs–I–Cs optical vibration, and longitudinal I–Pb–I frame, respectively (Figure 10b). While organic cations such as MA and FA in MAPbI_3_, MAPbBr_3_, and FAPbI_3_ did not show phonon vibrational modes interacting with the surrounding metal cations or halogen anions, the inorganic Cs cation led to Cs–I–Cs optical vibration. Additionally, the absorption peak appearance at 1.5 THz covers the intermediate 0.5–3 THz range, which presents a significant advantage over typical OHPs containing organic cations.

In Supporting Information, this study confirms the phase change from *γ*‐CsPbI_3_ into *δ‐*CsPbI_3_ over time caused by the structural degradation in ambient conditions and discusses the THz‐TDS experimental results for altered samples (Figure 10c).^[^ ^60^ ^]^ In conclusion, *δ‐*CsPbI_3_ demonstrates THz absorption despite exhibiting lower real conductivity (maximum of 35 S cm^−1^) compared to *γ*‐CsPbI_3_, We can expect that CsPbI_3_‐based THz sensors can maintain sufficient THz absorption despite the internal structural degradation.

## 5 Conclusion

**Figure 11:**
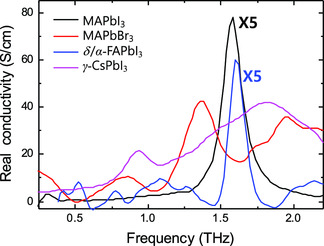
The measured THz‐wave absorptions in MAPbI_3_, MAPbBr_3_, *δ*/*α*‐FAPbI_3_, and *γ*‐CsPbI_3_ thin films. The THz‐wave absorptions of MAPbI_3_ and *δ*/*α*‐FAPbI_3_ are reduced in size by a factor of 5. Data replotted from data originally presented in refs. [55, 58, 60, 74].

We have investigated the THz absorption properties of the representative 3D OHP (AMX_3_) materials at RT. The consistent phonon vibrational modes observed in MAPbI_3_, MAPbBr_3_, FAPbI_3_, and CsPbI_3_ comprise Pb–I(or Br)–Pb buckling and Pb–I(Br)–Pb translational vibrations. However, the significant THz absorption property for each OHP material is caused by the different nature of each OHP material (**Figure** **11**). Furthermore, the absorption power of each OHP can be assessed through its absorption coefficient (cm^−1^) and real conductivity (S cm^−1^) (**Table** **1**).

**Table 1:** Maximum values of absorption coefficients and real conductivity in the range of 0.5–3.0 THz

–	Absorption coefficient [cm^−1^]	Real conductivity [S cm^−1^]
MAPbI_3_ (Solution)^[^ ^72^ ^]^	6000 (at 2.0 THz)	139
MAPbI_3_ (SVE)^[^ ^74^ ^]^	11 000 (at 1.58 THz)	254
MAPbBr_3_ (SVE)^[^ ^58^ ^]^	1700 (at 2.0 THz)	39
δ‐FAPbI_3_ (Single Crystal)^[^ ^58^ ^]^	433 (at 2.5 THz)	10
α‐FAPbI_3_ (Single Crystal)^[^ ^58^ ^]^	779 (at 1.8 THz)	18
δ‐ α ‐FAPbI_3_ (Single Crystal)^[^ ^58^ ^]^	1384 (at 2.0 THz)	32
γ‐CsPbI_3_^[^ ^60^ ^]^	1730 (at 1.8 THz)	40
δ‐CsPbI_3_^[^ ^60^ ^]^	1514 (at 1.4 THz)	35

Based on these findings, we discuss the direction for future research on THz sensor fabrication using OHP materials.

First, achieving coverage of the entire core frequency range of 0.5–3.0 THz with a single type of thin film is impractical. Therefore, future studies should create multilayer structures with different 3D OHPs to cover each desired frequency range. Notably, the investigated samples were fabricated using SVE, which facilitates multilayer fabrication. In the case of the solution‐prepared method, it is difficult to fabricate a multilayer structure because of the presence of solvents. On the other hand, the SVE method is based on the vacuum evaporation method which is more convenient for multilayer formation. However, it requires detailed postannealing conditions to avoid the formation of mixed‐hybrid perovskite structures (e.g., (MA, FA)(Pb, Sn)(Br, I)_3_) that have no significant THz absorption property.^[^ ^82^, ^83^ ^]^

Second, future material candidates should be based on Pb‐free perovskites because of the environmental problems associated with Pb. 2D OHP materials (e.g., MA_3_Sb_2_I_5_ and MA_3_Bi_2_I_5_) are an environmentally friendly alternative.^[^ ^6^, ^59^, ^84^, ^85^, ^86^ ^]^ These materials show enhanced surface stability compared to 3D OHPs, featuring high formation energy, which inhibits moisture erosion and suppresses ion migration.^[^ ^6^, ^59^, ^84^, ^85^, ^86^ ^]^ Furthermore, the 2D structure itself naturally provides a confinement effect that reduces electron–hole recombination, therefore aiding charge transport.^[^ ^6^, ^59^, ^84^, ^85^, ^86^ ^]^ Currently, the research on 2D OHPs is focused on single crystals to investigate various physical phenomena originating from the 2D structure and there is the lack of understanding of the atomic structure and chemical state of thin films. Therefore, there is an urgent need to investigate the fabrication methods and properties of the grain boundaries of 2D OHP thin films.

Finally, the investigation of material stabilities of the suggested candidates, such as multilayers using 3D and 2D OHPs, is necessary. THz sensors are operated at RT, and since we can assume a lack of temperature‐dependent problems in the materials, only the material itself poses a critical problem. In a multilayer structure, additionally, the interface stability must also be considered.

In summary, we have explored THz absorption properties in 3D OHP materials. From this short review, we found several issues such as increasing PA, solving Pb exclusion (Pb free), and improving material stability. Finally, we expect THz sensors based on OHP materials sooner that have various merits such as low unit cost, easy fabrication, high PA (<4000 cm^−1^ at 0.5–3.0 THz), and flexibility of device.

## Conflict of Interest

The authors declare no conflict of interest.
